# The role of platelet and endothelial GARP in thrombosis and hemostasis

**DOI:** 10.1371/journal.pone.0173329

**Published:** 2017-03-09

**Authors:** Elien Vermeersch, Frederik Denorme, Wim Maes, Simon F. De Meyer, Karen Vanhoorelbeke, Justin Edwards, Ethan M. Shevach, Derya Unutmaz, Hodaka Fujii, Hans Deckmyn, Claudia Tersteeg

**Affiliations:** 1 Laboratory for Thrombosis Research, IRF Life Sciences, KU Leuven Campus Kulak Kortrijk, Kortrijk, Belgium; 2 Laboratory of Immunology, National Institute of Allergy and infectious Diseases, Bethesda, MD, United States of America; 3 The Jackson Laboratory for Genomic Medicine, Farmington, CT, United States of America; 4 Chromatin Biochemistry Research Group, Combined Program on Microbiology and Immunology Research Institute for Microbial Diseases, Osaka University, Osaka, Japan; GERMANY

## Abstract

**Background:**

Glycoprotein-A Repetitions Predominant protein (GARP or LRRC32) is present on among others human platelets and endothelial cells. Evidence for its involvement in thrombus formation was suggested by full knockout of GARP in zebrafish.

**Objectives:**

To evaluate the role of GARP in platelet physiology and in thrombus formation using platelet and endothelial conditional GARP knock out mice.

**Methods:**

Platelet and endothelial specific GARP knockout mice were generated using the Cre-loxP recombination system. The function of platelets without GARP was measured by flow cytometry, spreading analysis and aggregometry using PAR4-activating peptide and collagen related peptide. Additionally, clot retraction and collagen-induced platelet adhesion and aggregation under flow were analyzed. Finally, in vivo tail bleeding time, occlusion time of the mesenteric and carotid artery after FeCl3-induced thrombosis were determined in platelet and endothelial specific GARP knock out mice.

**Results:**

Platelet specific GARP knockout mice had normal surface GPIb, GPVI and integrin αIIb glycoprotein expression. Although GARP expression was increased upon platelet activation, platelets without GARP displayed normal agonist induced activation, spreading on fibrinogen and aggregation responses. Furthermore, absence of GARP on platelets did not influence clot retraction and had no impact on thrombus formation on collagen-coated surfaces under flow. In line with this, neither the tail bleeding time nor the occlusion time in the carotid- and mesenteric artery after FeCl3-induced thrombus formation in platelet or endothelial specific GARP knock out mice were affected.

**Conclusions:**

Evidence is provided that platelet and endothelial GARP are not important in hemostasis and thrombosis in mice.

## Introduction

The endothelium of the vascular wall prevents thrombus formation by releasing different anticoagulant and antiplatelet factors such as nitric oxide and prostaglandin PGI_2_. Upon endothelial injury, platelets are recruited towards the exposed collagen in the subendothelial matrix. Platelets rapidly adhere and become activated, resulting among others in alpha-granule release and activation of integrin αIIbβ3. This leads to the recruitment of additional platelets and adhesive proteins, and promotes the pro-coagulant activity of both platelets and endothelial cells, culminating in the formation of a thrombus sealing the injured vessel. However, when this process is ill-controlled, this may lead to bleeding or the formation of occlusive thrombi resulting in ischemic cardiovascular events. Only limited pharmaceutical tools are available to control the thrombotic and hemostatic properties of platelets. Hence, significant efforts are made towards the identification of unknown platelet and endothelial receptors that could eventually become therapeutic targets.

The Bloodomics Consortium previously identified uncharacterized platelet receptors with a possible involvement in thrombosis and hemostasis [1,2]. Gene expression profiles of human megakaryocytes were compared with those of erythroblasts and different leukocytes using a whole genome microarray. Glycoprotein A Repetitions Predominant protein (GARP) also known as Leucine Rich Repeat Containing protein 32 (LRRC32), was one of the receptors newly identified to be present on the megakaryocyte lineage as well as on endothelial cells [3]. GARP is a 72-kDa glycoprotein that consists of a 13-amino acid cytoplasmic tail, a single transmembrane domain and 20 extracellular leucine rich repeats [4,5]. Besides megakaryocytes and endothelial cells, GARP was already known to be present on activated regulatory T cells (Tregs)[6–8], some fibroblast cell lines [9] and hepatic stellate cells [10]. The function of GARP is best elucidated for activated Tregs. Although the exact mechanism is not yet known, GARP expressed on activated Tregs plays a role in surface presentation of GARP/latent transforming growth factor (TGF)-β1 complexes and in the release of active TGF-β1 [11]. In platelets, GARP was shown to be co-expressed with latent TGF-β1 on the cell membrane [6]. Interestingly, involvement of GARP in thrombus formation was suggested in zebrafish, in which a defect in thrombus growth was observed in the absence of GARP [3]. Due to the generation of a full GARP knockout zebrafish, it was indistinguishable whether the obtained results could be explained by GARP expressed on platelets or on endothelial cells.

The present study aims to further investigate the role of GARP in thrombosis and hemostasis. Transgenic mice lacking GARP expression on platelets and megakaryocytes were generated using *Pf4*.*Cre* mice and on mice lacking GARP expression on endothelial cells using *Tie2*.*Cre* mice. Using these unique mouse strains, the contribution of GARP in thrombosis, hemostasis and thrombo-inflammatory processes was studied by in vitro platelet function assays, as well as in vivo bleeding, thrombosis and stroke models.

## Material and methods

### Mice

C57BL/6J mice were purchased from The Jackson Laboratory (Bar Harbor, ME). *Pf4* (courtesy W. Bergmeier, University of North Carolina, Chapel Hill, NC) and *Tie2* (courtesy M. Yanagisawa, Southwestern Medical School, Dallas, TX) promotor-driven *Cre* recombinase transgenic mice, and mice carrying the Garp^*fl*/fl^ alleles were described previously [12–14]. Exon 1 of Garp^*fl*/fl^ mice is flanked by *loxP* sites. Excision of exon 1 results in complete disruption of GARP expression. *Pf4* or *Tie2* conditional GARP knockout mice (cKO) were generated by first mating mice carrying the *Cre* allele driven by the *Pf4* or *Tie2* promotor with the Garp^*fl*/fl^ mice. Garp^*fl*/-^*Cre*^+/-^ mice were backcrossed with Garp^*fl*/fl^ mice to generate *Pf4* or *Tie2* specific *Garp* knockout mice (cKO, Garp^*fl*/fl^ *Cre*^+/-^) and littermate controls (Garp^*fl*/fl^ *Cre*^-/-^). In experiments, 8 to 12 weeks old mice were used unless noted otherwise.

### Polymerase Chain Reaction (PCR)

Genotypes for loxP, Cre driven by the Pf4 promotor and Cre driven by the Tie2 promotor were determined with PCR amplification of total genomic DNA obtained after NaOH extraction of ear samples, using three pairs of primers: 5’-GCAAAGCAGACGGTCATACA-3’, 5’-TCTGGAACTCAAGAGGCTGAG-3’; 5’-GTCCACAGCTGGTGTGGAGA–3’, 5’-GGCACCATAGATCAGGCGGT-3’ and 5’-CGCCGTAAATCAATCGATGAGTTGCTTC–3’, 5’-GATGCCGGTGAACGTGCAAAACAGGCTC-3’, respectively.

### Platelet preparation

Platelets were prepared according to standard procedures with minor modifications [15]. Citrated blood (5:1 vol/vol of blood: 3.8% sodium citrate) was taken via the retro-orbital sinus of mice anesthetized with 5% isoflurane in O2. Blood was diluted (1:1) with HEPES Tyrode buffer (145 mM NaCl, 2 mM KCl, 0.5 mM NaH2PO4, 5.5 mM glucose, 10 mM HEPES, 1 mM MgSO4, pH 7.4). Platelet rich plasma (PRP) was obtained via centrifugation at 80 g for 7 min at room temperature (RT). The remaining blood sample was then centrifuged during 6 min at 2000 g to obtain platelet poor plasma (PPP). For preparation of washed platelets, 1/10 ACD (85mM sodium citrate, 74mM citric acid, 44μM glucose) was added to the PRP and platelets were centrifuged at 360 g for 7 min. The pellet was resuspended in HEPES Tyrode buffer pH 6.5, and 1 μM PGE1 and 100 mU apyrase were added before subsequent centrifugation. The final pellet was resuspended in HEPES Tyrode buffer pH 7.4.

### Hematological analysis

Mice were anesthetized with 5% isoflurane/O2 and blood was taken from the retro-orbital sinus on EDTA (15:1 vol/vol of blood: 0.5M EDTA). Total blood count was performed using an automated cell counter (Hemavet 1700, Drew Scientific Group, Waterbury, CT). Platelet count, mean platelet volume, white blood cell (WBC), red blood cell (RBC), hemoglobin (Hb) and hematocrit (HCT) levels were analyzed.

### Platelet aggregometry

Platelet aggregometry was performed as described [16]. The PRP platelet count was normalized to 2.0 x 105 platelets/μL using HEPES Tyrode buffer. Platelet aggregation was monitored using light transmission in a Chrono-Log dual channel aggregometer (Kordia BV, Leiden, The Netherlands). PPP was used as a reference. Platelet aggregation was induced by adding 0.75, 2.5 or 5 μg/mL collagen-related peptide (CRP; courtesy RW Farndale, University of Cambridge, Cambridge, UK) or by adding 75, 125 or 250 μM protease activated receptor 4 activating peptide (PAR4-AP; AYPGKF, Bachem, Bubendorf, Switzerland).

### Flow cytometry

#### Platelets

Flow cytometry experiments were performed as described [17]. Briefly, citrated whole blood was diluted 1:20 in HBS buffer (10 mM HEPES, 150 mM NaCl, 1 mM MgSO4, 5 mM KCl, pH 7.4). Platelets in diluted whole blood were activated by adding 700 μM PAR4-AP or 20 μg/mL CRP during 20 min at RT before measuring the GARP expression. Platelet activation with 200 μM PAR4-AP or 2.5 μg/mL CRP was performed to measure the P-selectin expression on littermate and GARP cKO platelets.

Expression was determined using fluorescently-labeled antibodies against murine GPVI (JAQ1), CD42c (Xia.C3), P-selectin (Wug.E9), activated integrin αIIbβ3 (JON/A) (all from Emfret, Eibelstadt, Germany), GARP (REA 139; Miltenyi Biotec, Leiden, The Netherlands) and CD41 (MwReg30, Biolegend, London, UK). Antibody dilutions were used according to the manufacturer’s instructions. To measure platelet-fibrinogen binding, diluted blood was incubated for 20 min at RT with 50 μg/mL FITC-labeled human fibrinogen (Gentaur, Kampenhout, Belgium), PE labeled anti-CD41 (MwReg30 Biolegend) and 200 μM PAR4-AP or 2.5 μg/mL CRP. Samples were fixed (0.2% formaldehyde, 154 mM NaCl, pH 7.4) and analyzed on a FACSVerse flow cytometer (Becton, Dickinson and Company, Franklin Lakes, NJ).

#### Endothelial cells

Pieces from livers obtained from mice perfused with PBS buffer (137 mM NaCl, 2.7 mM KCl, 6.5 mM Na2HPO4, 1.5 mM KH2PO4) were digested using 2.5 mg/mL collagenase A and 20 U/mL DNase. A single cell suspension was made using a 70 μm nylon filter. GARP expression on endothelial cells was measured in the CD45-CD41-CD31+ cell population. Expression was determined using fluorescently-labeled antibodies against murine CD45 (30-F11, eBioscience, Vienna, Austria), CD41 (MwReg30), CD31 (390) and GARP (REA 139) (both from Miltenyi Biotec). Antibody dilutions were used according to the manufacturer’s instructions.

### Platelet spreading

Spreading of platelets was analyzed as previously described [18]. Briefly, coverslips were coated overnight with 200 μg/mL human fibrinogen (Sigma Aldrich, Diegem, Belgium). Coated coverslips were blocked with 1% BSA in PBS and rinsed with HEPES Tyrode buffer. Washed platelets were stimulated with 200 μM PAR4-AP or 2.5 μg/mL CRP during 5, 10 and 30 min at RT on coated coverslips and non-adhering platelets were washed using HEPES Tyrode buffer. Adherent platelets were fixed with 4% PFA and visualized with a Zeiss observed Z.1 inverted microscope (Carl Zeiss, Sliedrecht, The Netherlands). Quantification of platelets was performed by counting the platelets in different phases of activation: discoid (phase 1), only filopodia (phase 2), filopodia and lamellipodia (phase 3) and fully spread platelets (phase 4).

### Clot retraction

Clot retraction experiments were performed as previously described [18]. Briefly, one μL of erythrocyte suspension was added to 200 μL of citrated PRP in which platelet counts were normalized to 300,000 platelets/μL using PPP. Clot formation was induced with 20 mM CaCl2 and 5 U/mL thrombin (Sigma Aldrich) at 37°C. Clot retraction was followed over time. Every 2 min, a picture was taken with a digital camera. Clot size was analyzed using ImageJ (version 1.47, NIH, Bethesda, MD).

### Thrombus formation in flow chambers

Polydimethylsiloxane perfusion chambers were placed on glass coverslips creating a channel with a height of 75 μm and a width of 2 mm. The channels were coated with 100 μg/mL Horm collagen (abp, Epsom, UK) for one hour [19]. Heparinized (20 U/ml, LEO Pharma, Wilrijk, Belgium) whole blood was perfused over the coverslip at a shear rate of 1600 s-1 for 5 minutes. Before perfusion, blood was incubated with 5 μM Dioc6 (Invitrogen, Ghent, Belgium) during 10 min at 37°C. Thrombus formation was visualized for 5 min under an Eclipse TE200 inverted fluorescence microscope (Nikon, Tokyo, Japan) coupled to a Hamamatsu CCD camera (ORCA-R2, Hamamatsu Photonics, Hamamatsu, Japan). Aggregate formation was analyzed as mean percentage platelet coverage of the total area using ImageJ.

### Intravital microscopy of FeCl3 injured mesenteric and carotid blood vessels

Carotid artery thrombosis was induced as previously described [20]. Mice were anesthetized using a mixture of 2% isoflurane in O2. The blood flow in the right carotid artery was measured using a flow probe (Transonic TS420 perivascular flowmeter module, AD Instruments, Paris, France). Thrombus formation was induced by placing a Whatman filter paper saturated with 12% FeCl3 for 3 min on the surface of the carotid artery, upstream of the flow probe. The mesenteric thrombosis model was performed as previously described [21]. Mesenteric arteries of Xylazine/Ketamine anesthetized mice (5 weeks old) were exposed to a Whatman filter paper saturated with 10% FeCl3 for 2 min. Platelets were fluorescently labeled by intravenous injection of rhodamine 6G (1 μg/g body weight) (Invitrogen). Thrombus formation in the mesenteric artery was followed in real time using an Eclipse TE200 inverted fluorescence microscope (Nikon) under a 20X objective coupled to a Hamamatsu CCD camera (ORCA-R2, Hamamatsu Photonics) [21]. In both models, the occlusion time, defined by a lack of detectable blood flow after arterial injury, was recorded. The thrombi were followed up during 20 minutes to monitor the thrombus stability.

### Transient middle cerebral artery occlusion

Focal cerebral ischemia was induced in mice by 60 minutes transient middle cerebral artery occlusion (tMCAO) [20,22]. Mice were anesthetized with 2.5% isoflurane in O2. Following a midline skin incision in the neck, the proximal right common carotid artery and the external carotid artery were ligated, and a standardized silicon rubber-coated 6.0 nylon monofilament was advanced through the right internal carotid artery to occlude the origin of the right MCA. The intraluminal suture was left in situ for 60 minutes, after which the animals were re-anesthetized and the occluding monofilament was withdrawn to allow reperfusion. Twenty-three hours after reperfusion mice were euthanized to measure cerebral infarct volumes. Brains were quickly isolated and cut into 2-mm-thick coronal sections using a mouse brain slice matrix. The slices were stained with 2% 2,3,5-triphenyl-tetrazolium chloride to distinguish healthy tissue from unstained infarctions. Stained slices were photographed and infarct areas (white) were measured using Image J software. Edema-corrected infarct sizes were calculated by use of the following equation: Vcorrected = Vuncorrected x (1 - (Vi—Vc) / Vc) with Vi the volume of ipsilateral hemisphere and Vc the volume of the contralateral hemisphere.

### Ethics statement

Animal experiments were approved by the Institutional Animal Care and Use Committee of KU Leuven, Leuven, Belgium (Permit Number: P022-2012).

### Statistics

Results are presented as mean ± standard deviation (SD). Statistical significance was assessed using GraphPad Prism version 5.00 (GraphPad Software, San Diego, CA, USA). ANOVA statistics was used to measure the GARP expression on platelets. An unpaired t-test was performed to reveal differences between littermates and GARP cKO mice. P-values < 0.05 were considered significant.

## Results

### GARP is expressed on murine platelets and is increased after activation

**Fig 1:**
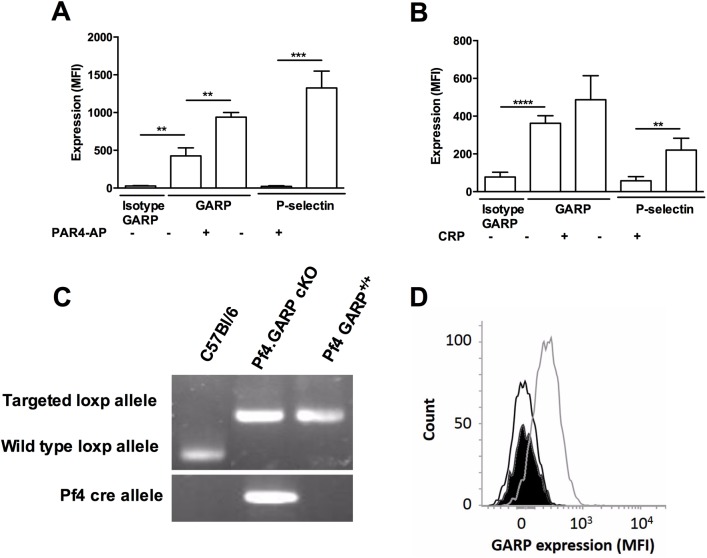
Generation of platelet specific GARP knockout mice. GARP and P-selectin expression on 700 μM PAR4-AP (**A**) or 20 μg/mL CRP (**B**) activated murine platelets. Mean fluorescence intensity (MFI) of anti-GARP-APC and anti-P-selectin CD62P-PE are shown. **(C)** Genotypic analysis of genomic DNA from control C57BL/6, littermates and platelet specific cKO mice. *Loxp* is 181 bp (fl/fl); wt allele is 111 bp, *Cre* 250 bp. (**D**) Phenotypic characterization of littermates and cKO mice using flow cytometry. Platelets were labeled with anti-CD41-FITC and anti-GARP-APC and a histogram was made based on APC intensity of CD41+ cells: isotype control (black line), littermates (grey line) and cKO (black).

Platelets isolated from C57BL/6J mice were stained for P-selectin and GARP using fluorescently-labeled antibodies. Flow cytometric analysis of stained platelets revealed that GARP is expressed on resting murine platelets. The resting status of the platelets is confirmed by the absence of P-selectin on the surface. Furthermore, when platelets were activated by PAR4-AP, not only P-selectin expression was increased, as expected, but interestingly, also the GARP expression was increased 2.2 fold (Fig 1A). Similarly, GARP expression levels on the platelet surface were increased 1.34 fold upon activation with CRP, although this increase was not significant (Fig 1B).

### Platelet specific GARP knockout mice have normal blood cell counts and normal levels of GPIb, GPVI and integrin αIIb glycoprotein on their platelet surface

The CMV promotor driven *Cre* recombinase was used to generate full *GARP* knockout mice. As however mice expressing the *Cre* recombinase and being homozygous for the deleted Garp allele had an embryonic lethal phenotype (S1 Fig), we next specifically knocked down GARP on platelets by site specific recombination using the *Pf4* promoter driven *Cre* recombinase to generate *GARP* cKO (*loxP*^+/+^ *Cre*^+/-^) and littermates (*loxP*^+/+^ *Cre* ^-/-^). The genotyping of C57BL/6 mice, littermates and GARP cKO mice demonstrates the presence of *loxP* sites (181 bp) and the *Cre* recombinase (250 bp) in the genomic DNA (Fig 1C). Correct recombination was confirmed by demonstrating the absence of GARP on platelets of *Pf4* specific *GARP* knockout mice by flow cytometry (Fig 1D). The platelets of *Pf4* specific *GARP* knockout mice did not show overall abnormalities as the platelet volume and expression levels of the surface glycoproteins GPIb, GPVI and integrin αIIb were unaltered. Additionally, no significant differences were found in platelet, WBC and RBC counts, and Hb and HCT levels between littermates and GARP cKO mice (Table 1). The platelet specific GARP knockout mice were born at the expected Mendelian inheritance, showed no discernible phenotype, were fertile and their body weights were comparable to controls.

**Table 1:** GARP cKO platelets are well formed and GARP cKO mice display unaltered hematological parameters.

	Pf4 GARP^+/+^	Pf4 GARP cKO	P-value
GPIb (MFI ± SD)	5445 ± 181	5345 ± 194	n.s.
GPVI (MFI ± SD)	527 ± 22	583 ± 31	n.s.
αIIb (MFI ± SD)	2.297 ± 1.099 x 10^4^	2.426 ± 0.765 x 10^4^	n.s.
PLT Vol. (fL)	5.52 ± 0.23	4.63 ± 0.25	n.s.
PLT X 10²/μL	684.5 ± 82	659.0 ± 75	n.s.
WBC X 10²/μL	7.11 ± 1.53	6.58 ± 2.22	n.s.
RBC X 10^6^/μL	8.95 ± 1.08	8.95 ± 1.11	n.s.
Hb (g/dL)	11.40 ± 1.07	11.43 ± 1.61	n.s.
HCT (%)	42.07 ± 4.95	42.97 ± 6.29	n.s.

Platelet receptors are quantified as mean fluorescence intensity (MFI) ± SD; n = 3; Platelet volume (PLT Vol.); platelet (PLT); white blood cell (WBC)and red blood cell (RBC) count, hemoglobin (Hb) and hematocrit (HCT) levels; data are expressed as mean ± SD; n = 10; n.s. non-significant.

### GARP deficiency in platelets does not affect platelet activation and spreading

**Fig 2:**
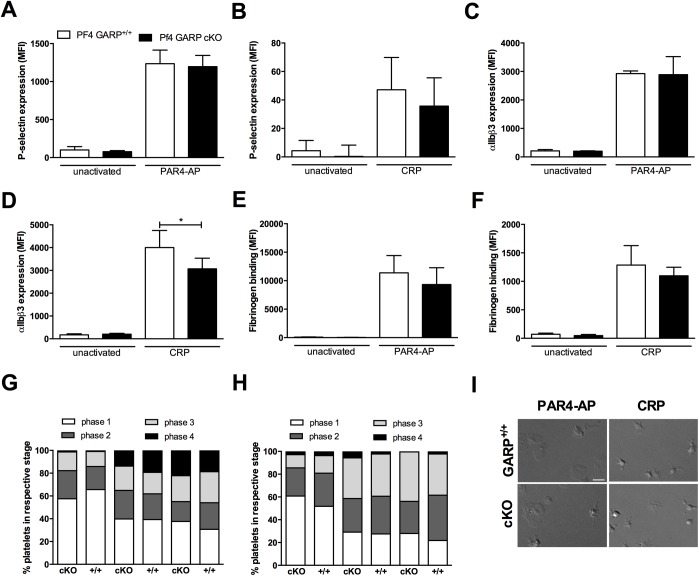
GARP expression is elevated upon platelet activation but does not affect outside-in and inside-out signaling. MFI of P-selectin (anti-CD62P-PE) (**A-B**), JON/A (anti-GPIIbIIIa-PE) (**C-D**) and fibrinogen binding (**E-F**) to unactivated or 200 μM PAR4-AP (**A-C-E**) or 2.5 μg/mL CRP (**B-D-F**) activated platelets of littermates and cKO mice are measured using flowcytometry. Platelets were gated based on CD41 (anti-CD41-FITC) expression. Graphs show mean ± SD, n = 3. Washed platelets of littermates and cKO mice were allowed to spread on 100 μg/mL fibrinogen while activated with 200 μM PAR4-AP (**G**) or 2.5 μg/mL CRP (**H**) during 5, 10 or 30 min. Platelets are divided in different phases of activation (1) round, (2) only filopodia, (3) filopodia and lamellipodia, (4) fully spread. (**I**) Representative images of platelet spreading; scale bars represent 5 μm.

Since the GARP expression on mouse platelets is increased after activation and as previous experiments in zebrafish suggested that GARP is implicated in thrombus formation [3], we next studied the effect of GARP deficiency on *in vitro* platelet activation. First, platelets from GARP cKO and littermates were stimulated with 200 μM PAR4-AP or 2.5 μg/mL CRP and activation was assessed via flow cytometry by measuring expression of P-selectin and activated integrin αIIbβ3, as well as fibrinogen binding. As expected, platelet activation with PAR4-AP (Fig 2A, 2C and 2E) and CRP (Fig 2B, 2D and 2F) resulted in increased P-selectin expression (Fig 2A and 2B), increased activation of integrin αIIbβ3 (Fig 2C and 2D) and an increased fibrinogen binding (Fig 2E and 2F). Absence of GARP on platelets did not affect P-selectin expression and fibrinogen binding (Fig 2A, 2B, 2E and 2F), however a slightly but significantly (*P* = 0.04) lower activation of integrin αIIbβ3 was observed in the cKO platelets compared to control platelets after stimulation with CRP (Fig 2D).

When platelet activation is initiated, platelets undergo morphologic changes as a result of actin skeleton reorganization. On fibrinogen-coated surfaces, this results in a transformation from discoid shaped- to fully spread platelets. To verify if GARP is involved in this process, platelets were stimulated with 200 μM PAR4-AP (Fig 2G) or 2.5 μg/mL CRP (Fig 2H). Filopodia and lamellipodia are formed in the GARP cKO mice to the same extent as in the littermate controls (representative images are shown in Fig 2I). All together, these results demonstrate that GARP is not involved in the activation of platelets although GARP expression is increased after activation.

### GARP deficiency in platelets does not affect platelet aggregation, clot retraction nor platelet adhesion on collagen

**Fig 3:**
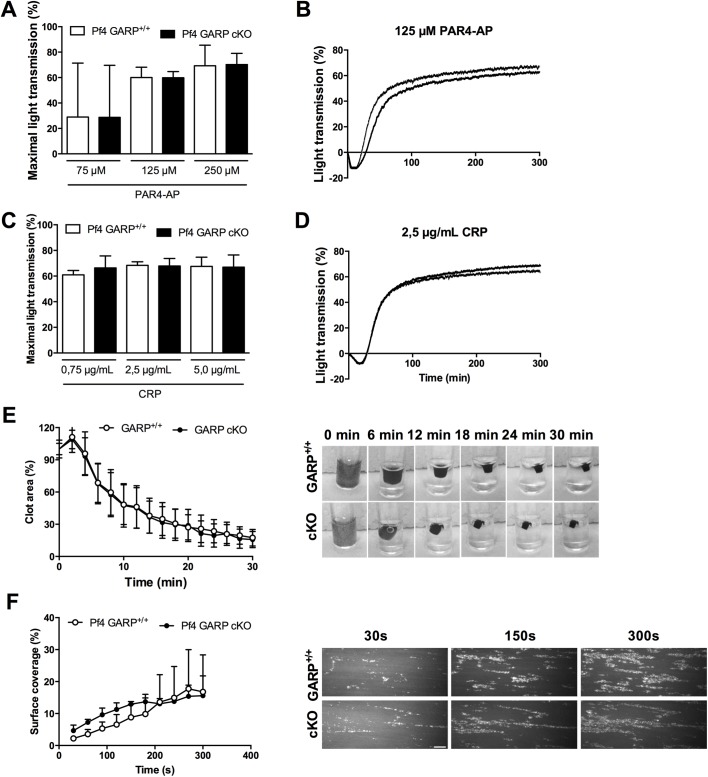
GARP deficiency in platelets does not affect platelet aggregation. Aggregation was induced in PRP of GARP^+/+^ and GARP cKO mice using 75, 125 or 250 μM PAR4-AP (**A**) and 0.75, 2.5 or 5 μg/mL CRP (**C**). Maximal light transmission was calculated and graphed of at least 3 individual experiments. Representative curves for 125 μM PAR4-AP (**B**) and 2.5 μg/mL CRP (**D**) induced aggregations in GARP^+/+^ (grey) and GARP cKO (black) are shown. (**E**) Clot retraction of PRP supplemented with erythrocytes was induced using 5 U/mL thrombin and 20 mM CaCl_2_ in GARP^+/+^ littermates and GARP cKO. Pictures were made every two minutes and clot area was analyzed using ImageJ. Representative pictures are shown on the right. Measured clot area is presented on the right; n = 3. (**F**) Whole blood of GARP cKO mice and GARP^+/+^ littermates was perfused over a Horm collagen (100 μg/mL) coated coverslip at a shear rate of 1600 s^-1^. Thrombus formation was visualized during 5 min and surface coverage was quantified using ImageJ; n = 3. Representative pictures of thrombus formation under flow are shown on the right; scale bars represent 50 μm.

To further assess the potential effect of GARP on platelet function, we performed aggregations with PRP from GARP cKO mice and littermates. Platelet aggregation was induced via a G protein-coupled receptor-mechanism by PAR4-AP or via Tyr-phosphorylation by CRP using a low, intermediate and high concentration of each agonist. GARP deficient platelets aggregated in a similar fashion compared to their littermate controls in response to both PAR4-AP (Fig 3A) and CRP (Fig 3B). Representative tracings of aggregations induced by 125 μM PAR4-AP and 1.25 μg/mL CRP are shown in Fig 3B and 3D, respectively. In line with platelet activation, no difference in aggregation was observed between platelets with and without GARP (Fig 3B and 3D).

As we did not observe a role for GARP in either platelet activation, spreading or aggregate formation, we questioned whether GARP might be involved in the next phase of thrombus formation being clot retraction. After an aggregate is formed, platelets will contract their cytoskeleton. This mechanism affects the fibrin network bound to integrin αIIbβ3 and reduces the volume of the clot. To initiate thrombus formation and subsequent clot retraction, thrombin and CaCl_2_ were added to PRP of littermates and GARP cKO mice. The clot retraction was followed for 30 min and a gradual decrease in clot volume was measured (Fig 3E; quantification on left, representative images on right). Again, GARP cKO mice did not show an altered clot volume over time compared to littermates, which indicates that platelet GARP is not involved in clot retraction.

All experiments performed thus far were performed under static or rotational flow conditions and did not incorporate shear stress. Therefore, we next performed perfusions over collagen-coated surfaces to examine the potential role of platelet GARP in thrombus formation under flow. Platelets in whole blood were labeled with Dioc6, and then perfused at an arterial shear rate of 1600 s^-1^. Platelets adhered readily to collagen and subsequent platelet aggregate formation was followed for 5 min. The surface coverage was quantified and demonstrated no difference between GARP cKO mice and littermates (Fig 3F; quantification on left, representative images on right), indicating that GARP does not influence platelet adhesion and aggregation under arterial shear stress.

### Platelet specific GARP cKO mice display normal tail bleeding, FeCl_3_-induced thrombosis and infarct volume after ischemic stroke

**Fig 4:**
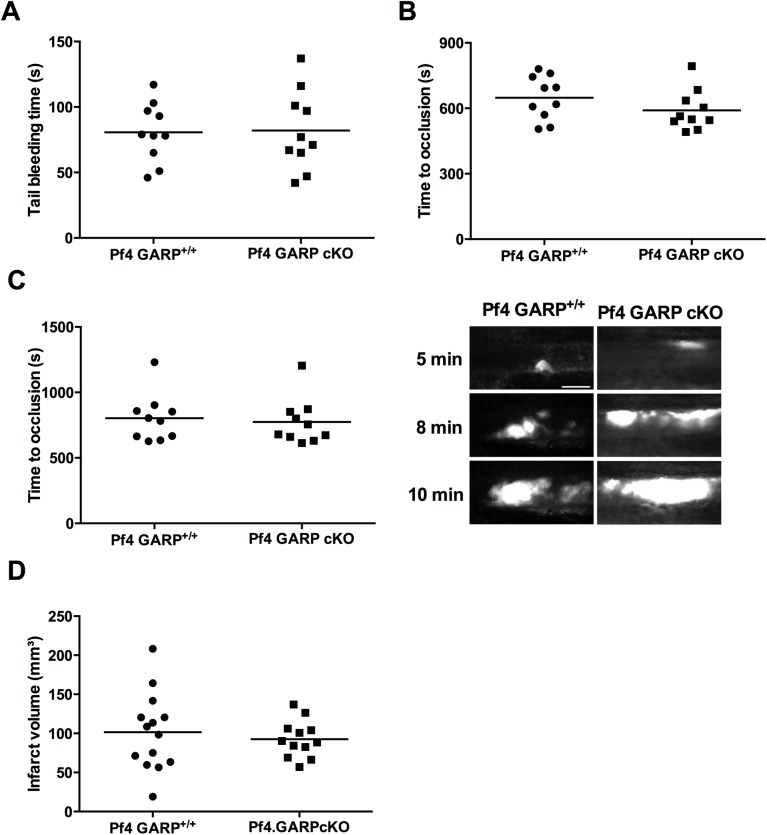
Unaltered bleeding time and thrombus formation in *Pf4* specific GARP cKO mice. (**A**) 2 mm of the tail tip was dissected and tail bleeding times were measured (n = 10). (**B**) Carotid artery was injured using topical application of 12% FeCl_3_ and blood flow was monitored using a flow probe until flow stopped due to the formation of an occlusive thrombus (n = 10). (**C**) Mesenteric arteries were injured using 10% FeCl_3_ and thrombus formation was followed using intravital microscopy until full occlusion was reached (n = 10). Representative pictures after 5, 8 and 10 min of mesenteric thrombus formation are shown on the right. Scale bars represent 50 μm. **(D)** The right middle cerebral artery of littermate mice (n = 14) and platelet specific GARP knockout mice (n = 12) was occluded during 60 min and reperfusion was allowed during 23 hours.

As the ex vivo assays could not indicate a role for GARP in platelet function, we next investigated whether GARP is involved in physiological hemostasis and thrombosis using in vivo mouse models. First, we performed a tail clip bleeding time assay (Fig 4A), which revealed that the average bleeding time of littermates was not significantly different from that of the GARP cKO mice. In parallel, we assessed the involvement of platelet-specific GARP in arterial thrombus formation in well-established carotid and mesenteric FeCl_3_-induced arterial thrombosis models [21]. In the carotid artery, occlusive thrombus formation was monitored using a Doppler flow probe for up to 20 min post injury. In this model both littermates and GARP cKO mice did form occlusive thrombi at a similar rate (Fig 4B). In mesenteric arteries, occlusive thrombus formation was measured by real time imaging of the thrombus after fluorescent labeling of the platelets. Complete vessel occlusion was comparable for GARP cKO mice and and littermates (Fig 4C; quantification on left, representative images on right). In both the carotid and the mesenteric artery thrombosis model, no differences were observed in embolization or clot stability between GARP cKO mice and littermates.

Platelets play a major role in stroke progression and platelet receptors such as GPIb and GPVI are known to be involved in the thrombo-inflammatory process [23]. To investigate whether GARP on platelets contributes to the ischemia/reperfusion process, the tMCAO model was performed in littermate and platelet specific GARP knockout mice. The infarct volumes after 60 min of tMCAO and 23 hours of reperfusion were measured but did not differ (Fig 4D). Together, these results are in line with the ex vivo results, and all together indicate that GARP expressed on platelets is neither required for thrombosis, hemostasis nor thrombo-inflammation.

### GARP is expressed on mouse endothelium and is absent on *Tie2* specific GARP knockout mice

Overall, our results demonstrate that a deficiency of GARP on mouse platelets does not affect thrombus formation and therefore could not explain the thrombotic phenotype observed in the GARP deficient zebrafish. We therefore generated endothelium specific GARP knockout mice as GARP is also highly expressed on human endothelial cells [3]. Moreover, endothelial cells are important in the formation and stabilization of thrombi as they express amongst others adhesion receptors and secrete von Willebrand factor, but also limit platelet activation by formation of PGI_2_ and NO.

**Fig 5:**
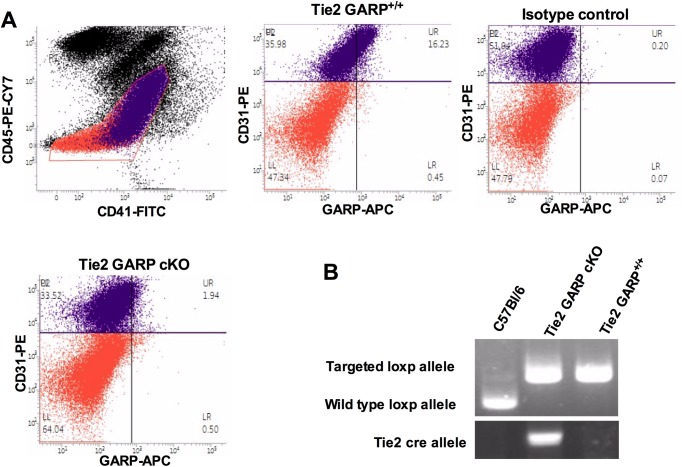
Generation of endothelial specific GARP knockout mice. (**A**) Phenotypic characterization of littermates and cKO mice using flow cytometry. Single cells were labeled with anti-CD45-PeCy7, anti-CD31-PE, anti-CD41-FITC and anti-GARP-APC and a histogram was made based on APC intensity of CD45-CD31+CD41- population. Isotype control (black line), littermates (grey line) and cKO (black). (**B**) Genotypic analysis of genomic DNA from control C57BL/6, littermates and endothelial specific cKO mice. *Loxp* is 181 bp (fl/fl); wt allele is 111 bp, *Cre* 450 bp.

First, GARP expression on murine endothelial cells was measured. Single liver cells were isolated and incubated with antibodies against CD31 (endothelial marker), CD45 (leukocyte marker) and CD41 (platelet marker), as well as antibodies against GARP. As shown in Fig 5A, GARP indeed is expressed on murine CD31+CD45-CD41- endothelial cells. Isotype matched antibodies were used as a negative control for the anti-GARP monoclonal antibodies and demonstrated no positive staining. The genotyping of C57BL/6 mice, littermates and endothelial specific GARP knockout mice demonstrates the presence of *loxP* sites (181 bp) and the *Cre* recombinase (450 bp) in the genomic DNA (Fig 5B). Correct recombination was confirmed by demonstrating the absence of GARP on CD31+CD45-CD41- single endothelial liver cells by flow cytometry (Fig 5A). This confirms the successful generation of *Tie2* specific *GARP* knockout mice. The mice were born at the expected Mendelian frequencies, showed no discernable phenotype, were fertile and the body weights were comparable between the endothelial specific GARP knockout mice and littermate controls.

### Endothelial GARP is not important in thrombus formation or thrombo-inflammation

**Fig 6:**
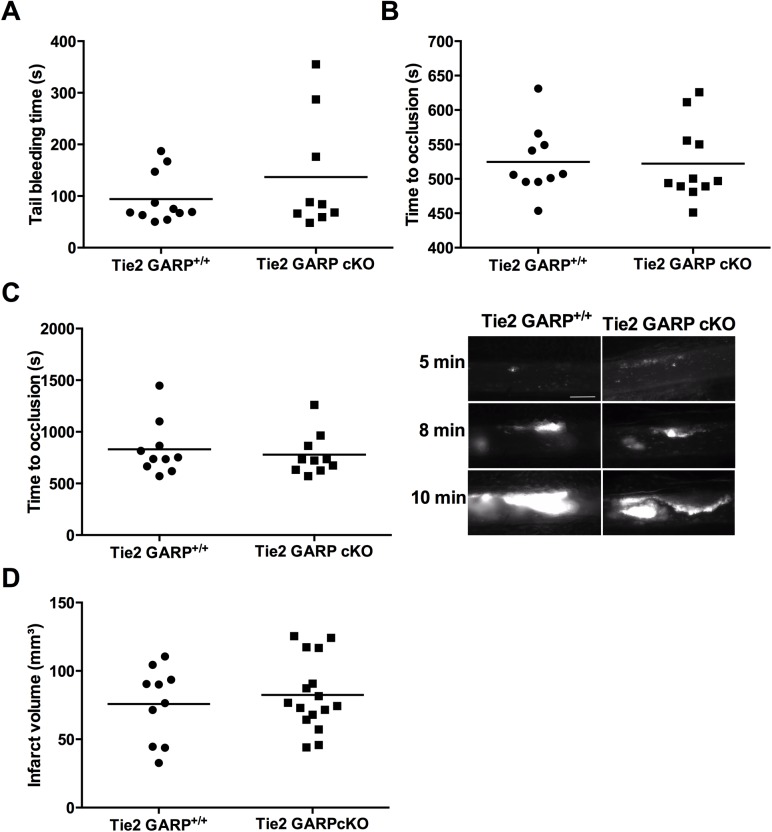
Unaltered bleeding time and thrombus formation in *Tie2* specific GARP cKO mice. (**A**) 2 mm of the tail tip was dissected and tail bleeding times were measured (controls: n = 11; cKO: n = 9). (**B**) Carotid artery was injured using topical application of 12% FeCl_3_ and blood flow was monitored using a flow probe until flow stopped due to the formation of an occlusive thrombus (controls: n = 10; cKO: n = 11). (**C**) Mesenteric arteries were injured using 10% FeCl_3_ and thrombus formation was followed using intravital microscopy until full occlusion was reached (controls: n = 10; cKO: n = 11). Representative pictures after 5, 8 and 10 min of mesenteric thrombus formation are shown on the right. Scale bars represent 50 μm. **(D)** The right middle cerebral artery of littermate mice (n = 10) and endothelium specific GARP knockout mice (n = 16) was occluded during 60 min and reperfusion was allowed during 23 hours.

Similar to the platelet specific GARP cKO mice, the tail bleeding time did not differ between the endothelium specific GARP cKO and the littermates (Fig 6A). In the carotid artery, occlusive thrombus formation was monitored and both littermates and GARP cKO mice did form occlusive thrombi at a similar rate (Fig 6B). Accordingly, complete mesenteric artery occlusion was comparable for the GARP cKO mice and littermates (Fig 6C; quantification on left, representative images on right). In both the carotid and the mesenteric artery thrombosis model, no differences were observed in embolization or clot stability between GARP cKO mice and littermates. To investigate whether GARP on endothelial cells contributes to the ischemia/reperfusion process, the tMCAO model was performed in littermate and endothelium specific GARP knockout mice. The infarct volumes after 60 min of tMCAO and 23 hours of reperfusion were comparable for the endothelium specific GARP knockout mice and controls (Fig 6D). In conclusion, these results strongly suggest that GARP expressed on murine platelets as well as on murine endothelial cells is neither important for thrombosis, hemostasis nor thrombo-inflammation.

## Discussion

Thrombus formation is a dynamic process in which platelet receptors and components of the endothelium play a crucial role in the adhesion, aggregation and stabilization of the growing thrombus. A comparative transcript study identified 279 novel platelet membrane receptors of which ESAM, BAMBI, DCBLD2, GARP and ANTXR2 were selected for further investigation in zebrafish (with αIIb as a positive control) [3]. In the zebrafish study, the targeted gene was completely down regulated in the whole genome by applying antisense morpholino oligonucleotides. Based on the effects seen in a laser induced thrombosis model in the caudal artery of the respective gene deficient zebrafish, a function for all but ANTXR2 membrane receptors in thrombosis was proposed. However, as the different receptors are not only expressed in the megakaryocyte lineage, but also on e.g. endothelial cells, some types of leukocytes and erythroblasts, the relevant cell responsible for the effect in thrombosis was not defined. Therefore, there is a need for follow-up studies in which the targeted gene is selectively removed from specific cells. In the zebrafish study, BAMBI was identified as a positive regulator of thrombosis. Salles-Crawley et al. further investigated its function in thrombosis and hemostasis using chimeric mice. The previously observed results in zebrafish were confirmed [15], with a thrombus-stabilizing role for BAMBI expressed in endothelium whereas platelet BAMBI has no role in thrombosis and hemostasis. Also the contribution of ESAM, suggested to be a negative regulator of thrombus formation in zebrafish, was further elucidated. A mouse study confirmed the function of ESAM in thrombus growth and stability, and herein suggests a critical role for platelet ESAM [24]. In the current study, we set out to determine whether GARP would affect thrombus formation in mice. As full *Garp* knockout mice had an embryonic lethal phenotype, we generated both platelet and endothelium specific GARP knockout mice. The expression of GARP on human platelets [3,6] and endothelial cells (3) was already reported previously and we now demonstrated that GARP is also expressed on murine platelets and endothelium.

Platelet specific GARP knockout mice were generated, allowing comprehensive assessment of GARP deficiency on platelets. No differences were measured in ex vivo α-granule release, platelet adhesion, spreading and clot retraction. However, a significantly decreased integrin αIIbβ3 activation was measured in GARP cKO platelets after activation with CRP. Nevertheless, this small difference in activation did not influence the other more physiological integrin αIIbβ3 related ex vivo assays as no differences were observed in fibrinogen binding, platelet spreading, clot retraction and aggregation. Accordingly, in vivo tail bleeding time or the time to occlusion of the carotid or mesenteric artery after FeCl_3_-induced thrombosis and the tMCOA stroke model did not show any difference between platelet specific GARP cKO mice and littermate controls. Furthermore similar results were obtained in the endothelium specific GARP knockout mice, strongly indicating that neither platelet nor endothelial GARP is involved in thrombosis and hemostasis.

The hypothesis of this study was based on the functional screening of putative novel platelet membrane proteins from the comparative whole-genome expression analysis by the induction of arterial thrombosis following morpholino oligonucleotide knockdown in zebrafish [3]. The zebrafish is a well-characterized animal model to study human disease phenotypes, more specifically in the field of megakaryopoiesis and hemostasis due to the high conservation of platelet functions such as thrombocyte adhesion and aggregation. Therefore the zebrafish was postulated to be a good functional model to select genes involved in thrombosis [25–27]. The results demonstrated that the two tested morpholinos used to downregulate GARP in zebrafish gave diverse results in both time to adhesion and the surface area of the thrombus formed after laser injury of the caudal artery. Moreover, negative control morpholinos did not demonstrate similar results between the different investigated receptors [3]. Therefore, further investigation using murine thrombosis models was necessary to clarify these results. In this study, knocking out GARP specifically in mouse platelets or endothelial cells could not confirm the positive thrombus regulating phenotype of GARP in zebrafish. This inconsistency may be explained by the variation measured in the zebrafish study or by differences between zebrafish and mouse species. Zebrafish thrombocytes are nucleated, the GPIb receptor is differently expressed in zebrafish and downregulation of other proteins in zebrafish, such as hepsin using morpholino oligonucleotides also resulted in inconsistent results with the gene knockout in mice [28]. Taken together, this indicates that thrombosis models in zebrafish are not always a good predictor for gene function in mice.

In this study, we showed that the GARP full knockout mice were not viable, but endothelium and platelet specific conditional GARP knockout mice were born and did not show any overt phenotype. Also CD4 T cell specific GARP knockout mice [14] and Foxp3 specific GARP knockout mice (unpublished results) were demonstrated to be viable. This suggests that GARP may be involved in the mouse embryonal development by its ability to bind latent TGF-β [6,8,14,29]. This however, is beyond the scope of our current study.

An alternative role for GARP was found in the release of active TGF-β [30]. On human platelets, co-expression of GARP and the latency associated peptide, which noncovalently associate with mature dimeric latent TGF-β, was reported [6,29]. The α-granules of platelets are the major source of latent TGF-β, a cytokine which is released upon platelet activation. Although most of the released latent TGF-β is bound to the latent TGF-β binding protein and contributes to the TGF-β1 levels in the circulation, 40% of the latent TGF-β is present in a platelet-rich thrombus [31–33]. The specific contribution of TGF-β to thrombus formation however, is not thoroughly elucidated. TGF-β by itself does not induce platelet aggregation [32,34], but seems to have a slightly activating effect when used in combination with different platelet agonists, such as collagen, U46619, ADP and SFLLRNP [34,35]. As Cuende et al. showed that inhibitory anti-GARP antibodies can block the release of active TGF-β on Treg, GARP may also be involved in the gradual release of active TGF-β on platelets and endothelium [11]. Consequently, GARP may be involved in the targeted delivery of TGF-β in wound repair, in particular in angiogenesis. Further studies will have to elucdiate the potential role of platelet or endothelium GARP in these processes.

## Supporting information

S1 Fig Full GARP knockout mice are not viable. Mice with the *Cmv* promotor driven *Cre* recombinase were crossed with Garp^*fl*/fl^ mice in the first generation. Garp^*fl*/-^*Cre*^+/-^ mice were backcrossed with Garp^*fl*/fl^ mice to generate full *Garp* knockout mice. (A) Genotypic analysis of genomic DNA from neonates from the second generation, *Floxed* allele is 670 bp (fl/fl); WT allele is 610 bp, *Cre* 200 bp. WT: wild type, Floxed: *Garp* allele is surrounded by *Loxp* sites, Loxp: *Garp* allele is deleted and 1 *Loxp* site remains (B) percentage born neonates are given for each possible genotype.
(TIFF)
