# Supplementary figures and images for: The role of platelet and endothelial GARP in thrombosis and hemostasis
Source: PLoS One. 2017 Mar 9;12(3):e0173329. doi: 10.1371/journal.pone.0173329 (PMC5344406; doi:10.1371/journal.pone.0173329)

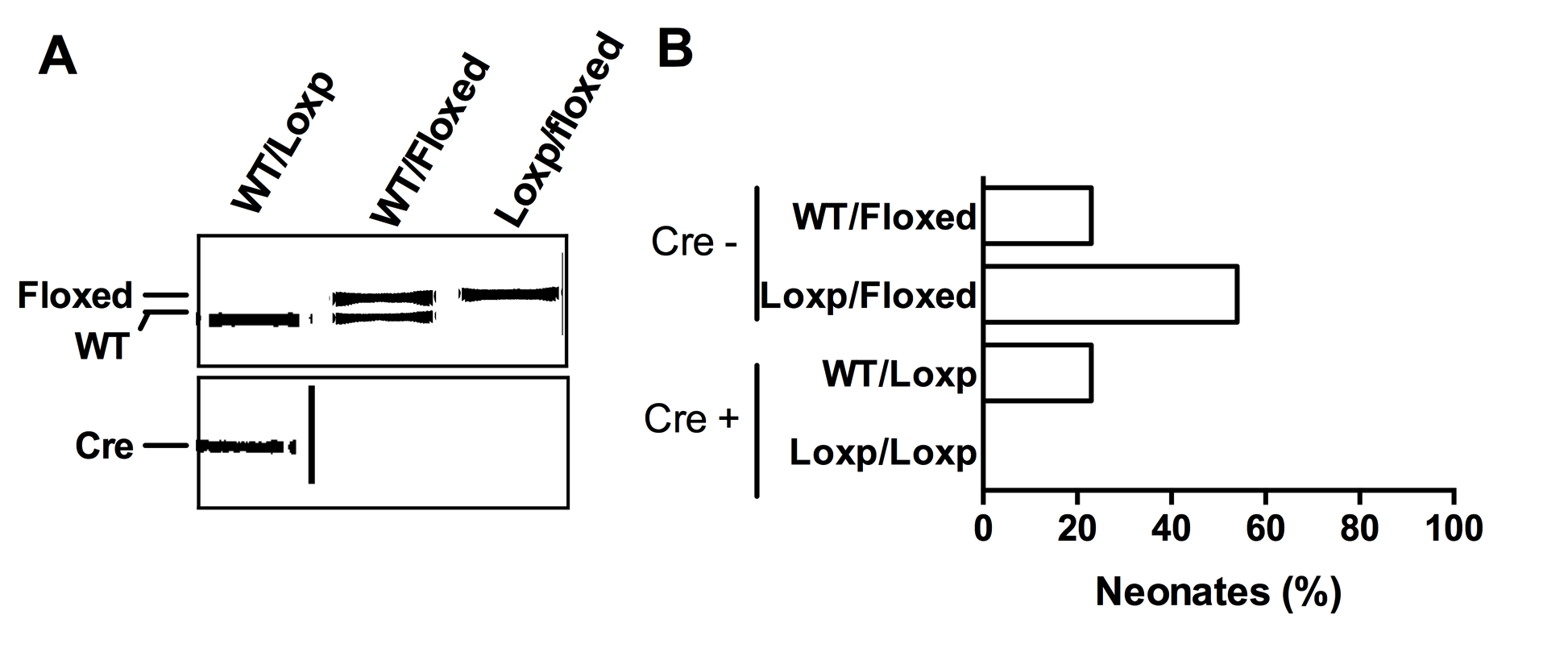

Supplement: S1 Fig — Mice with the Cmv promotor driven Cre recombinase were crossed with Garpfl/fl mice in the first generation. Garpfl/-Cre+/- mice were backcrossed with Garpfl/fl mice to generate full Garp knockout mice. (A) Genotypic analysis of genomic DNA from neonates from the second generation, Floxed allele is 670 bp (fl/fl); WT allele is 610 bp, Cre 200 bp. WT: wild type, Floxed: Garp allele is surrounded by Loxp sites, Loxp: Garp allele is deleted and 1 Loxp site remains (B) percentage born neonates are given for each possible genotype. (TIFF) [file pone.0173329.s001.tiff]
